# Mental Health Disorders and Pain in Patients Undergoing Head and Neck Free Flap Surgery

**DOI:** 10.1002/oto2.70105

**Published:** 2025-04-07

**Authors:** Kelly L. Vittetoe, Marina Aweeda, Lily Gao, Christopher Naranjo, Liping Du, Xiaoke Feng, Wenda Ye, Alexander J. Langerman, Kyle Mannion, James L. Netterville, Eben L. Rosenthal, Robert J. Sinard, Michael C. Topf, Sarah L. Rohde, Alexander H. Gelbard, Melanie D. Hicks

**Affiliations:** ^1^ Vanderbilt Medical Center Department of Otolaryngology Nashville Tennessee USA; ^2^ Vanderbilt University School of Medicine Nashville Tennessee USA; ^3^ Vanderbilt University Department of Biostatistics Nashville Tennessee USA

**Keywords:** head and neck cancer, mental health disorders, microvascular reconstruction, postoperative pain

## Abstract

**Objective:**

Determine relationships between pain and mental health disorders (MHDs) in patients undergoing microvascular free flap reconstruction for head and neck cancer (HNC).

**Study Design:**

Retrospective cohort.

**Setting:**

Tertiary Care Institution in the Southeastern United States.

**Methods:**

Clinical data were manually abstracted from digital health records to obtain demographic, MHD, clinical outcomes, and pain data for HNC patients who underwent free flap reconstruction from 2017 to 2023. Univariate and multivariable regression analyses were performed to delineate relationships between MHDs and postoperative pain.

**Results:**

The study cohort comprised 283 patients. Ninety‐four patients (33%) had preoperative MHDs, which were more common in women (42% vs 30%, *P* = .04) and in patients with chronic pain (53% vs 32%, *P* < .01). Preoperative opioid use (*P* = .03) and preoperative MHD (*P* = .03) were predictive of higher postoperative day (POD) 5 pain score. Thirty‐three patients (11.7%) were diagnosed with a new MHD postoperatively, and 58 patients (20.5%) were started on a new long‐term psychiatric medication postoperatively. POD1 pain score was predictive of the need for a new psychiatric medication postoperatively (odds ratio [OR] = 1.27, 95% CI: 1.05‐1.56, *P* = .02).

**Conclusion:**

Postoperative pain and MHDs are independently predictive of one another in patients with HNC undergoing microvascular free flap reconstruction. Higher POD5 pain is predicted by the presence of preoperative MHD, and the need for a new psychiatric medication postoperatively is predicted by higher POD1 pain. HNC surgeons should align themselves with psychiatrists, social workers, and other allied fields to meet the complex mental health needs of their patients both preoperatively and postoperatively.

In patients with head and neck cancer (HNC), mental health disorders (MHDs) are highly prevalent and may influence postoperative outcomes.^1^, ^2^, ^3^ Studies have reported up to 40% of HNC patients are affected by MHDs, most commonly major depressive disorder (MDD).^4^, ^5^, ^6^ This may even be an underestimate, as a recent study using patient‐reported surveys found that one in five patients with HNC has an undiagnosed MHD preoperatively.^7^ Pretreatment depression has been shown to independently predict poor functional outcomes and decreased survival in patients with HNC.^5^, ^8^, ^9^, ^10^, ^11^, ^12^ Moreover, the suicide mortality rate in patients with HNC is known to be 2 to 3 times higher than that of the general population.^13^

It is well known that pain and MHDs are intricately connected, particularly in the setting of cancer. Several prior studies in cancer of all types have shown that comorbid MHDs are associated with worse patient‐reported pain scores.^14^, ^15^, ^16^, ^17^ Although opioids are a well‐established treatment for cancer‐related pain,^18^, ^19^ HNC patients have an exceptionally high rate of chronic opioid use (COU) after treatment.^20^, ^21^ Such high rates of opioid use portend the risk of physical dependence and subsequent addiction, which has important psychosocial implications.^22^ Conversely, uncontrolled pain may also have a psychological impact, which in turn influences outcomes.^23^ Notably, surgery for HNC has been shown to lead to worse psychological outcomes compared to nonsurgical treatment modalities for HNC.^24^

Despite the widespread use of microvascular free flaps for patients with locally advanced HNC, there is a relative scarcity of studies focused on psychological outcomes following these surgeries.^11^, ^24^, ^25^, ^26^ Free flap surgeries for head and neck reconstruction are often disfiguring and carry a significant burden of morbidity, with substantial impact on communication and other social activities such as eating and drinking.^12^, ^27^, ^28^ No study has yet assessed both preoperative and postoperative pain in conjunction with preoperative and postoperative MHDs in HNC patients undergoing microvascular free flap reconstruction.

To investigate this knowledge gap, our group conducted a retrospective study examining MHDs and perioperative pain in HNC patients undergoing microvascular free flap reconstruction. We hope that our study allows for quality improvement interventions to target mental health and postoperative pain outcomes in this patient population.

## Methods

This retrospective cohort study was approved by the Vanderbilt University Medical Center Institutional Review Board (IRB #190168). An institutional database of patients who underwent free flap reconstruction within the otolaryngology department at a tertiary care center was reviewed, and patients aged ≥18 years who underwent surgery from November 2017 through January 2023 were included. Patients undergoing surgery for primary oncologic resection, recurrent oncologic resection, or other conditions such as osteoradionecrosis, fistula, or afunctional larynx were included in the study.

A manual chart review of the Electronic Medical Record (EMR) was conducted to obtain data on patient demographics, oncologic history, preoperative chronic pain diagnoses, preoperative and postoperative mental health diagnoses, narcotic and psychiatric medications, surgery details, postoperative pain scores, and clinical outcomes. Diagnoses of chronic pain were determined based on the presence of International Classification of Diseases (ICD)‐10 codes for chronic pain (G89.2, G89.3) or duration of narcotic prescription for 3 months or longer preoperatively.^29^ MHDs were identified by ICD‐10 codes. If there was free text information in clinic notes that reported an existing preoperative MHD not cataloged in the problem list, this was also recorded as an MHD.

Pain scores were documented on a 0 to 10 numeric rating scale, with 0 representing no pain and 10 representing “the worst pain imaginable.”^30^ Opioid administration on each postoperative day (POD) was measured in morphine milliequivalents (MME). A widely used measure of narcotic dosage, MME is calculated by converting an opioid medication dosage into milligrams of morphine, thereby standardizing dosage across medications. Previously established ratios were used to convert each medication into corresponding MME,^31^ allowing comparisons to be made between patients regardless of opioid medication type.

All data were stored in a secure RedCap database.^32^, ^33^ Statistical comparisons between groups were made with Mann‐Whitney and Kruskal‐Wallis tests for continuous variables as indicated as well as Fisher's exact and chi‐square tests for categorical variables as indicated. Linear regression models were constructed for continuous outcomes in multivariable analysis, and logistic regression models were used for binary outcomes. Covariates previously reported in the literature to associate with MHDs or postoperative pain were included in the models. An alpha value of .05 was set to indicate statistical significance for all tests. Missing data were few and not imputed. All analyses were performed using R version 4.4.0 and GraphPad Prism version 10.0.2.

## Results

### Demographics and Oncologic Characteristics

Two hundred eighty‐three patients met the inclusion criteria for the study. Median age was 66 years (interquartile range [IQR]: 58‐72), and 196 patients (70%) were men. Oral cavity tumors accounted for 77% of all tumors, laryngeal/hypopharyngeal 16%, and oropharyngeal 6.5%. The most common anatomic subsite was oral tongue (n = 60, 22%), followed by mandibular gingiva/alveolus (n = 48, 17%).

One hundred sixty‐six patients (59%) had advanced stage disease, characterized by pathologic T stage of 3 or 4. One hundred five patients (37%) had clinical nodal metastases preoperatively. One hundred eleven patients (40%) had undergone prior treatment for their cancer—either chemotherapy (n = 17; 6.1%), radiation (n = 27, 9.7%), or both (n = 67, 24%) (Table 1).

**Table 1:** Demographics, Oncologic, and Surgical Data

	Total N = 283
Age, y; median (IQR)	66 (58‐72)
Gender	
Male	196 (70%)
Female	87 (30%)
Primary tumor site	
Oral cavity	154 (77%)
Oropharynx	23 (11%)
Hypopharynx/larynx	24 (12%)
T stage	
0, no disease^a^	48 (17%)
1	15 (5.3%)
2	54 (20%)
3	60 (22%)
4	106 (39%)
N stage	
0	174 (61%)
1	39 (14%)
2	46 (17%)
3	20 (7.4%)
Treatment history	
None	172 (60%)
Chemotherapy	17 (6%)
Radiation	27 (10%)
Chemoradiation	67 (24%)
Flap type	
RFFF, fasciocutaneous	130 (47%)
RFFF, osteocutaneous	48 (17%)
ALT	65 (23%)
Fibula	15 (5.4%)
Latissimus dorsi	10 (3.6%)
Scapula	9 (3.2%)
Other	6 (2.1%)

Abbreviations: ALT, anterolateral thigh; IQR, interquartile range; RFFF, radial forearm free flap.

^a^ T0 included osteoradionecrosis, fistula, prior flap failure, and an afunctional larynx.

### Surgical Details

Soft tissue‐only flaps were more commonly performed (74%) compared to bony flaps (26%). The most frequent microvascular free flap was the fasciocutaneous radial forearm free flap (n = 130, 47%), followed by the anterolateral thigh free flap (n = 65, 23%). Additional surgical details are shown in Table 1.

### Preoperative Chronic Pain and MHDs

One hundred eleven patients (40%) had a preoperative diagnosis of chronic pain and were on narcotic medication before surgery. Among patients on preoperative narcotics, the median daily opioid dose measured in MME was 45 (IQR: 30‐71.25) (Table 2).

**Table 2:** Preoperative Chronic Pain and Mental Health Disorders

	Total N = 283
Chronic pain diagnosis	111 (40%)
Narcotic prescription	111 (40%)
Morphine milliequivalents; median (IQR)	45 (30‐71.25)
Mental health disorder	94 (33%)
Depression	65 (23%)
Anxiety	51 (18%)
PTSD/panic disorder	4 (1.4%)
Bipolar disorder	3 (1.1%)
Other	8 (2.8%)
Psychiatric medication	87 (31%)
SSRI	41 (14%)
Benzodiazepine	32 (40%)
Atypical antidepressant	20 (7.1%)
SNRI	11 (3.9%)
Quetiapine	5 (1.8%)
Aripiprazole	2 (0.7%)
Tricyclic antidepressant	2 (0.7%)
Lithium	1 (0.4%)

Abbreviations: IQR, interquartile range; PTSD, posttraumatic stress disorder; SNRI, serotonin‐norepinephrine reuptake inhibitor; SSRI, selective serotonin reuptake inhibitor.

Ninety‐four patients (33%) had a diagnosed MHD preoperatively. The most common MHD was depression (n = 65, 23%), followed by anxiety (n = 51, 18%). Twenty‐seven patients (9.5%) had comorbid depression and anxiety. Eighty patients of the 94 with a diagnosed MHD were on a psychiatric medication preoperatively (85%). The most common medication was a selective serotonin reuptake inhibitor (SSRI) (n = 41, 15%), followed by benzodiazepine (n = 32, 40%) and atypical antidepressant (n = 20, 7.1%) (Table 2).

Women were more likely than men to have a preoperative MHD (42% vs 30%, *P* = .037). Patients with preoperative chronic pain were also more likely to have a preoperative MHD (53% vs 32%, *P* < .001) (Table 3). As outlined above, chronic pain was determined based on the presence of the ICD‐10 code or duration of narcotic prescription for 3 months or longer preoperatively.^29^

**Table 3:** Preoperative Characteristics by MHD

	Preoperative MHD	No preoperative MHD	
	N = 94	N = 189	*P*‐value
Men	58 (62%)	138 (74%)	.04*
Women	36 (38%)	49 (26%)	
Current smoker	21 (23%)	45 (24%)	.78
Prior smoker	43 (46%)	89 (48%)	
Never smoker	29 (31%)	50 (27%)	
Current alcohol	27 (29%)	50 (27%)	.77
Prior alcohol	26 (28%)	46 (25%)	
No alcohol	40 (43%)	87 (48%)	
PO intake	60 (67%)	144 (77%)	.06
Preoperative chronic pain	50 (53%)	61 (32%)	<.01*
Preoperative narcotic rx	48 (51%)	63 (33%)	<.01*
T1	6 (7.6%)	9 (5.8%)	.37
T2	23 (29%)	31 (20%)	
T3	18 (23%)	42 (27%)	
T4	32 (41%)	74 (47%)	
No prior treatment	57 (61%)	115 (61%)	.99
Prior chemo/radiation	37 (39%)	74 (39%)	

Abbreviations: MHD, mental health disorder; PO, per oral.

* *P* < .05.

### Postoperative Pain and Opioid Requirement

**Figure 1:**
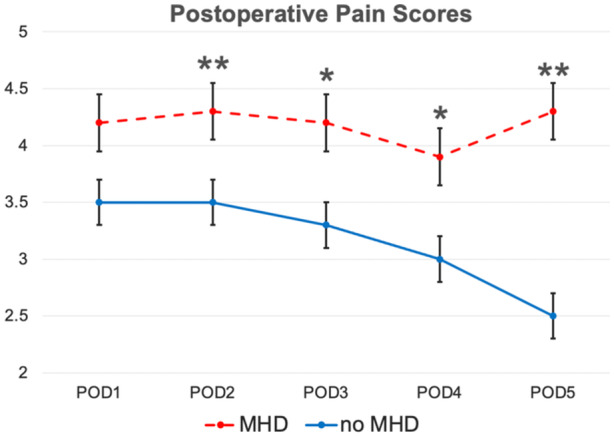
Postoperative pain scores in patients with and without a preoperative mental health disorder (MHD). Error bars reflect the standard error of mean. POD, postoperative day. *P < .05; **P < .01.

Overall, the median pain score on POD1 was 4.0, which decreased to 2.9 on POD5. In patients with a preoperative MHD, the mean pain score on POD1 was 4.2, compared to 3.5 in patients without a preoperative MHD (*P* = .100). On POD5, the mean pain score in the preoperative MHD group was 4.3, compared to 2.5 in patients without preoperative MHD (*P* = .003) (Figure 1). There was no difference between flap types in pain scores across POD1 (*P* = .626) through POD5 (*P* = .760). Likewise, there was no difference across cancer subsites in postoperative pain (*P* = .260) or MME requirement (*P* = .170).

**Figure 2:**
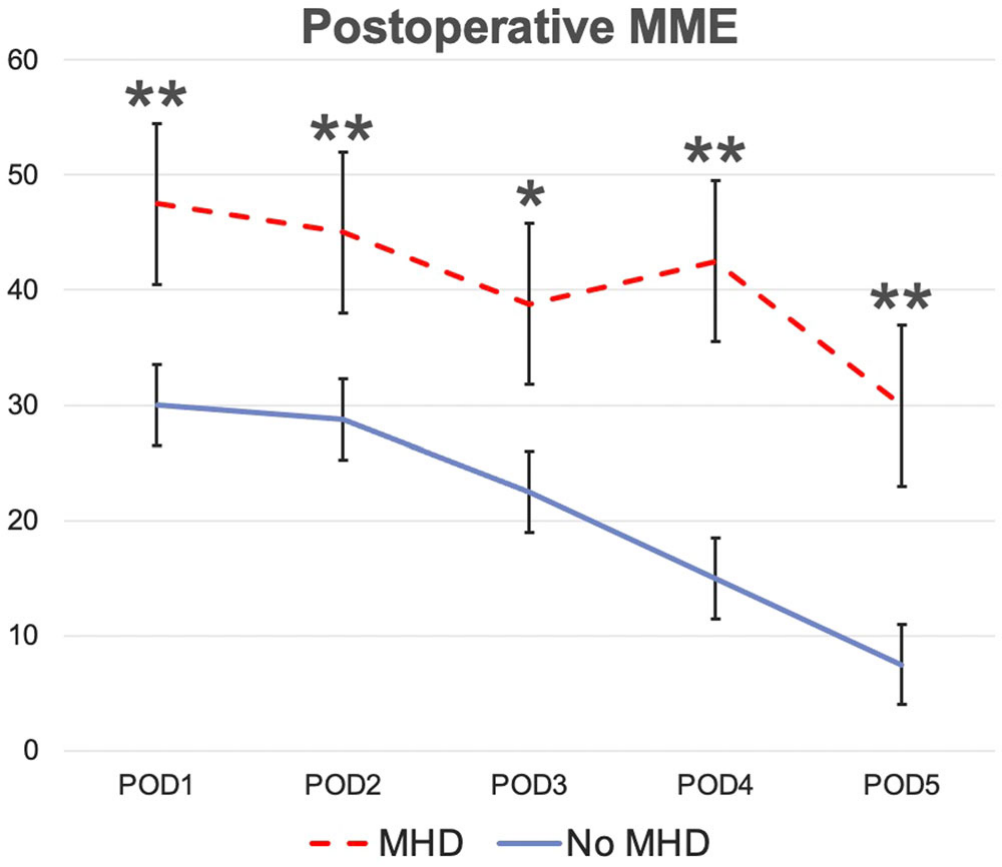
Postoperative morphine milliequivalents (MME) in patients with and without preoperative mental health disorder (MHD). Error bars reflect the standard error of mean. POD, postoperative day. *P < .05; **P < .01.

Mean MME per day for patients with a preoperative MHD was 47.5, compared to 30 in patients without preoperative MHD (*P* = .008). There was a significant difference in MME between groups on each POD (Figure 2).

### Postoperative MHDs

In total, 115 patients (41%) had a postoperative MHD diagnosis, and 114 (40%) were on psychiatric medication (Table 4). Thirty‐three patients (11.7%) were diagnosed with a new or different MHD postoperatively. For 29 patients (10.2%), this was their first MHD diagnosis. Furthermore, 58 patients (20.5%) were started on a new or different psychiatric medication postoperatively, either for a new or existing MHD.

**Table 4:** Postoperative Mental Health Disorders (MHDs)

	Total N = 283
New/different MHD	33 (12%)
New/different psychiatric medication	58 (20%)
Total with any MHD	115 (41%)
Total on any psychiatric medication	114 (40%)

### Multivariable Regression

To better elucidate predictive factors for increased postoperative pain, a multivariable linear regression model was used. Both preoperative narcotic (*β* = 1.72, *P* = .03) and preoperative MHD (*β* = 1.54, *P* = .03) were predictive of higher POD5 pain scores (Table 5).

**Table 5:** Multivariable Linear Regression for Postoperative Day 5 Pain

	Estimate	Standard error	*t*‐Value	*P*‐value
Gender (male)	0.28	0.44	0.64	.52
Smoking				
Current	−0.25	0.55	−0.45	.65
Prior	−0.24	0.47	−0.51	.61
Alcohol				
Current	−0.34	0.47	−0.73	.47
Prior	−0.42	0.49	−0.84	.40
Prior HNS	−0.05	0.42	−0.13	.91
Treatment history				
Chemotherapy	1.25	0.87	1.44	.15
Radiation	0.44	0.68	0.65	.52
Chemoradiation	0.40	0.52	0.78	.44
T stage				
0	0.38	1.00	0.38	.70
2	0.01	0.86	0.01	.99
3	0.10	0.87	0.12	.91
4	−0.27	0.82	−0.33	.74
Chronic pain	−0.31	0.83	−0.37	.71
Preoperative narcotic	1.77	0.81	2.18	.03*
Preoperative MHD	1.56	0.72	2.16	.03*
STSG	0.43	0.68	0.63	.53
Tracheostomy	0.34	0.52	0.67	.51

Abbreviations: HNS, head and neck surgery; MHD, mental health disorder; STSG, split‐thickness skin graft.

* *P* < .05.

Subsequently, to identify factors predictive of requiring a new psychiatric medication postoperatively, a multivariable logistic regression model was used. Only the POD1 pain score was predictive of the need for a new psychiatric medication postoperatively (odds ratio [OR] = 1.27, 95% CI: 1.05‐1.56, *P* = .02) (Table 6).

**Table 6:** Multivariable Logistic Regression for New Psychiatric Medication

	Odds ratio	Confidence interval (95%)	*P*‐value
Gender (male)	0.72	(0.34‐1.54)	.39
Smoking			
Current	2.12	(0.75‐6.23)	.16
Prior	2.18	(0.88‐5.77)	.10
Alcohol			
Current	0.96	(0.39‐2.29)	.92
Prior	1.20	(0.23‐1.59)	.32
Treatment history			
Chemo	0.61	(0.09‐2.58)	.56
Radiation	0.27	(0.04‐1.08)	.10
Chemoradiation	0.62	(0.25‐1.48)	.29
Tracheostomy	1.18	(0.56‐2.51)	.67
Chronic pain	0.99	(0.23‐3.92)	.99
Preoperative narcotic	1.72	(0.43‐7.37)	.45
POD1 mean pain	1.27	(1.05‐1.56)	.02*
POD5 mean pain	0.92	(0.76‐1.10)	.38

Abbreviation: POD, postoperative day.

* *P* < .05.

## Discussion

Patients with HNC have a high prevalence of MHDs compared to the general population, and this discrepancy becomes even greater for patients who undergo microvascular free flap surgery. It has been well‐established in prior studies that chronic pain and MHDs are intimately associated.^14^, ^15^, ^16^, ^17^ The present study redemonstrates this association in a cohort of patients with HNC undergoing microvascular free flap reconstructive surgery. Our study confirms the bidirectional and predictive relationship between pain and psychiatric comorbidities; higher POD5 pain is predicted by the presence of preoperative MHD, and the need for a new psychiatric medication postoperatively is predicted by higher POD1 pain.

The prevalence of preoperative MHDs in our study cohort (33%) approaches the upper limit of what has been reported in the literature.^1^, ^2^, ^3^, ^4^, ^5^, ^6^ The elevated prevalence of MHDs observed in our cohort may be related to the highly rural catchment area of our institution. Prior studies have found that the prevalence of depression in patients with HNC is higher in rural settings, which may be due to the relative scarcity of mental health care available in rural areas.^7^, ^34^ Moreover, the suicide rate in patients with HNC is higher for those who reside in rural compared to metropolitan or urban areas.^13^ This discrepancy has been attributed to correlations with social isolation, access to lethal means of suicide (eg, firearms), and greater susceptibility to the financial impacts of HNC care.^13^ At our tertiary care facility in the Southeast, patients commute from many surrounding states and often reside in rural counties. Screening for MHDs in high‐volume HNC practices, especially those serving rural populations, should therefore be prioritized.

Another important finding from the present study is the substantial number of patients who were diagnosed with a new MHD postoperatively—approximately 10% of the study cohort. It is difficult to determine whether these new diagnoses reflect a newly developed MHD or the delayed diagnosis of a preexisting MHD, perhaps exacerbated by treatment. It has been reported that patients with first‐onset MHD following oncologic surgery have worse mortality compared to those with a preoperative MHD.^35^ It is therefore important that surgeons are aware of this risk factor and appropriately screen for MHDs in the postoperative period as vigilantly as in the preoperative period.

Although screening is a critical first step, screening alone is insufficient. High‐quality screening for MHDs must be coupled with timely intervention or referral to an allied mental health professional to meet the needs of HNC patients with MHDs.^36^, ^37^ Additionally, as described above, the presence of preoperative MHD predicts higher postoperative pain scores. Therefore, particular attention should be paid to pain control in patients with known MHDs.

There are several important limitations to this study. First, this study was a retrospective chart review. We did not include screening measures that are commonly used in the clinical setting to diagnose depression and anxiety. We relied on ICD‐10 coding for the presence of MHDs, which carries an inherent risk of inaccuracy. An additional limitation is the time frame over which data were collected. We did not establish a formal cutoff point at which we stopped capturing new MHDs or psychiatric medication initiation. Therefore, it is possible that we overestimate the prevalence of patients who develop a new MHD as a result of their surgery and cancer treatment. However, we would argue that in the setting of a highly morbid surgery with lasting impacts on a patient's appearance, functionality, and communication, new MHDs developing in the years after surgery are likely tied in some way to a patient's journey through HNC. Finally, we do not comprehensively delve into some of the confounding factors for pain and mental health, including social support, coping mechanisms, and substance use disorders. We acknowledge the importance of these factors and regret that the retrospective design of our study did not allow for an adequate evaluation of these factors. This will be a key area of investigation for future studies.

Our results suggest that future studies should investigate the effect of improved pain control as well as mental health interventions postoperatively on the development of MHDs. If higher POD1 pain scores predict the need for a new psychiatric medication postoperatively, it stands to reason that improved postoperative pain control may have a positive impact on mental health outcomes. Future prospective studies to determine the impact of nonopioid adjuncts (eg, lidocaine and ketamine infusions, peripheral nerve blocks) on both pain control and postoperative MHDs are warranted.

In summary, mental health and postoperative pain are inextricably related. Each factor independently predicts the other. To capitalize on the interconnectedness of MHDs and pain in a positive way, we can intervene on both pain and mental health in the preoperative and postoperative settings. Modalities for psychosocial support and pharmacologic management of MHDs in patients with HNC are plentiful,^38^, ^39^ and head and neck oncologic surgeons should align themselves with psychiatrists, psychologists, social workers, and other allied fields to meet the complex mental health needs of their patients.

## Author Contributions

**Kelly L. Vittetoe**, study design, data collection/analysis, manuscript composition; **Marina Aweeda**, data collection, manuscript revision; **Lily Gao**, data collection, manuscript revision; **Christopher Naranjo**, data collection, manuscript revision; **Liping Du**, data analysis, manuscript revision; **Xiaoke Feng**, data analysis, manuscript revision; **Wenda Ye**, data collection, manuscript revision; **Alexander J. Langerman**, clinical expertise, manuscript revision; **Kyle Mannion**, clinical expertise, manuscript revision; **James L. Netterville**, clinical expertise, manuscript revision; **Eben L. Rosenthal**, clinical expertise, manuscript revision; **Robert J. Sinard**, clinical expertise, manuscript revision; **Michael C. Topf**, data analysis, clinical expertise, manuscript revision; **Sarah L. Rohde**, study oversight, data analysis, clinical expertise, manuscript revision; **Alexander H. Gelbard**, study oversight, data analysis, clinical expertise, manuscript revision; **Melanie D. Hicks**, study design/oversight, data analysis, clinical expertise, manuscript revision.

## Disclosures

### Competing interests

None.

### Funding source

None.

## References

[1] Huang RW , Chang KP , Marchi F , et al. The impact of depression on survival of head and neck cancer patients: a population‐based cohort study. Front Oncol. 2022;12:871915. 10.3389/fonc.2022.871915 PMC9453493 36091181

[2] Mäkitie AA , Alabi RO , Pulkki‐Råback L , et al. Psychological factors related to treatment outcomes in head and neck cancer. Adv Ther. 2024;41(9):3489‐3519. 10.1007/s12325-024-02945-3 39110309 PMC11349815

[3] Gao J , Tseng CC , Barinsky GL , et al. Exploratory analysis on the association of mental health disorders with in‐hospital postoperative complications and mortality in head and neck cancer surgery. Head Neck. 2021;43(10):3022‐3031. 10.1002/hed.26791 34180571

[4] Lee JH , Ba D , Liu G , Leslie D , Zacharia BE , Goyal N . Association of head and neck cancer with mental health disorders in a large insurance claims database. JAMA Otolaryngol Head Neck Surg. 2019;145(4):339‐344. 10.1001/jamaoto.2018.4512 30816930 PMC6481424

[5] Kim SA , Roh JL , Lee SA , et al. Pretreatment depression as a prognostic indicator of survival and nutritional status in patients with head and neck cancer. Cancer. 2016;122(1):131‐140. 10.1002/cncr.29693 26371775

[6] Sehlen S , Lenk M , Herschbach P , et al. Depressive symptoms during and after radiotherapy for head and neck cancer. Head Neck. 2003;25(12):1004‐1018. 10.1002/hed.10336 14648859

[7] Pichardo PFA , Desiato VM , Hellums RN , Altman KW , Purdy NC , Haugen T . Depression and anxiety in patients with head and neck cancer undergoing free flap reconstruction. Am J Otolaryngol. 2024;45(1):104044. 10.1016/j.amjoto.2023.104044 37734365

[8] Lazure KE , Lydiatt WM , Denman D , Burke WJ . Association between depression and survival or disease recurrence in patients with head and neck cancer enrolled in a depression prevention trial. Head Neck. 2009;31(7):888‐892. 10.1002/hed.21046 19309726

[9] Zimmaro LA , Sephton SE , Siwik CJ , et al. Depressive symptoms predict head and neck cancer survival: examining plausible behavioral and biological pathways. Cancer. 2018;124(5):1053‐1060. 10.1002/cncr.31109 29355901 PMC5821545

[10] Balachandra S , Eary RL , Lee R , et al. Substance use and mental health burden in head and neck and other cancer survivors: a National Health Interview Survey analysis. Cancer. 2022;128(1):112‐121. 10.1002/cncr.33881 34499355

[11] de Graeff A , de Leeuw JR , Ros WJ , Hordijk GJ , Blijham GH , Winnubst JA . Pretreatment factors predicting quality of life after treatment for head and neck cancer. Head Neck. 2000;22(4):398‐407. 10.1002/1097-0347(200007)22:4<398::AID-HED14>3.0.CO;2-V 10862025

[12] Dunne S , Mooney O , Coffey L , et al. Psychological variables associated with quality of life following primary treatment for head and neck cancer: a systematic review of the literature from 2004 to 2015. Psycho‐Oncology. 2017;26(2):149‐160. 10.1002/pon.4109 26918648

[13] Osazuwa‐Peters N , Barnes JM , Okafor SI , et al. Incidence and risk of suicide among patients with head and neck cancer in rural, urban, and metropolitan areas. JAMA otolaryngol Head Neck Surg. 2021;147(12):1045‐1052. 10.1001/jamaoto.2021.1728 34297790 PMC8304170

[14] Gauthier LR , Dworkin RH , Warr D , et al. Age‐related patterns in cancer pain and its psychosocial impact: investigating the role of variability in physical and mental health quality of life. Pain Med. 2018;19(4):658‐676. 10.1093/pm/pnx002 28340045

[15] Unseld M , Zeilinger EL , Fellinger M , et al. Prevalence of pain and its association with symptoms of post‐traumatic stress disorder, depression, anxiety and distress in 846 cancer patients: a cross sectional study. Psycho‐Oncology. 2021;30(4):504‐510. 10.1002/pon.5595 33210393 PMC8049050

[16] Spiegel D , Sands S , Koopman C . Pain and depression in patients with cancer. Cancer. 1994;74(9):2570‐2578. 10.1002/1097-0142(19941101)74:9<2570::AID-CNCR2820740927>3.0.CO;2-3 7923013

[17] Laird BJA , Boyd AC , Colvin LA , Fallon MT . Are cancer pain and depression interdependent? A systematic review. Psycho‐Oncology. 2009;18(5):459‐464. 10.1002/pon.1431 18942659

[18] Paice JA , Bohlke K , Barton D , et al. Use of opioids for adults with pain from cancer or cancer treatment: ASCO guideline. J Clin Oncol. 2023;41(4):914‐930. 10.1200/JCO.22.02198 36469839

[19] WHO guidelines for the pharmacological and radiotherapeutic management of cancer pain in adults and adolescents. World Health Organization. 2018. Accessed September 11, 2024. http://www.ncbi.nlm.nih.gov/books/NBK537492/ 30776210

[20] Hinther A , Rasool A , Nakoneshny SC , et al. Chronic opioid use following surgery for head and neck cancer patients undergoing free flap reconstruction. J Otolaryngol Head Neck Surg. 2021;50(1):28. 10.1186/s40463-021-00508-y 33892825 PMC8066487

[21] Sethi RKV , Panth N , Puram SV , Varvares MA . Opioid prescription patterns among patients with head and neck cancer. JAMA Otolaryngol Head Neck Surg. 2018;144(4):382‐383. 10.1001/jamaoto.2017.3343 29522065 PMC5876851

[22] Pergolizzi JV , Magnusson P , Christo PJ , et al. Opioid therapy in cancer patients and survivors at risk of addiction, misuse or complex dependency. Front Pain Res. 2021;2:2. 10.3389/fpain.2021.691720 PMC8915703 35295520

[23] Gureje O , Von Korff M , Simon GE , Gater R . Persistent pain and well‐being: a World Health Organization Study in Primary Care. JAMA. 1998;280(2):147‐151. 10.1001/jama.280.2.147 9669787

[24] Henry M , Rosberger Z , Ianovski LE , et al. A screening algorithm for early detection of major depressive disorder in head and neck cancer patients post‐treatment: longitudinal study. Psycho‐Oncology. 2018;27(6):1622‐1628. 10.1002/pon.4705 29532541

[25] Pierre CS , Dassonville O , Chamorey E , et al. Long‐term quality of life and its predictive factors after oncologic surgery and microvascular reconstruction in patients with oral or oropharyngeal cancer. Eur Arch Otrhinolaryngol. 2014;271(4):801‐807. 10.1007/s00405-013-2592-z 23771320

[26] Bozec A , Demez P , Gal J , et al. Long‐term quality of life and psycho‐social outcomes after oropharyngeal cancer surgery and radial forearm free‐flap reconstruction: a GETTEC prospective multicentric study. Surg Oncol. 2018;27(1):23‐30. 10.1016/j.suronc.2017.11.005 29549900

[27] Wu YS , Lin PY , Chien CY , et al. Anxiety and depression in patients with head and neck cancer: 6‐month follow‐up study. Neuropsychiatr Dis Treat. 2016;12:1029‐1036. 10.2147/NDT.S103203 27175080 PMC4854266

[28] Rhoten BA , Murphy B , Ridner SH . Body image in patients with head and neck cancer: a review of the literature. Oral Oncol. 2013;49(8):753‐760. 10.1016/j.oraloncology.2013.04.005 23683468

[29] Treede RD , Rief W , Barke A , et al. A classification of chronic pain for ICD‐11. Pain. 2015;156(6):1003‐1007. 10.1097/j.pain.0000000000000160 25844555 PMC4450869

[30] Breivik H , Borchgrevink PC , Allen SM , et al. Assessment of pain. Br J Anaesth. 2008;101(1):17‐24. 10.1093/bja/aen103 18487245

[31] Dowell D , Ragan KR , Jones CM , Baldwin GT , Chou R . CDC clinical practice guideline for prescribing opioids for pain—United States, 2022. MMWR Recomm Rep. 2022;71:1‐95. 10.15585/mmwr.rr7103a1 PMC9639433 36327391

[32] Harris PA , Taylor R , Thielke R , Payne J , Gonzalez N , Conde JG . Research electronic data capture (REDCap)—a metadata‐driven methodology and workflow process for providing translational research informatics support. J Biomed Inf. 2009;42(2):377‐381. 10.1016/j.jbi.2008.08.010 PMC2700030 18929686

[33] Harris PA , Taylor R , Minor BL , et al. The REDCap consortium: building an international community of software platform partners. J Biomed Inf. 2019;95:103208. 10.1016/j.jbi.2019.103208 PMC7254481 31078660

[34] Adamowicz JL , Christensen A , Howren MB , et al. Health‐related quality of life in head and neck cancer survivors: evaluating the rural disadvantage. J Rural Health. 2022;38(1):54‐62. 10.1111/jrh.12571 33720456 PMC8477149

[35] Zhu J , Fang F , Sjölander A , Fall K , Adami HO , Valdimarsdóttir U . First‐onset mental disorders after cancer diagnosis and cancer‐specific mortality: a nationwide cohort study. Ann Oncol. 2017;28(8):1964‐1969. 10.1093/annonc/mdx265 28525559

[36] Hahn EE , Munoz‐Plaza CE , Pounds D , et al. Effect of a community‐based medical oncology depression screening program on behavioral health referrals among patients with breast cancer: a randomized clinical trial. JAMA. 2022;327(1):41‐49. 10.1001/jama.2021.22596 34982119 PMC8728610

[37] Andersen BL , Lacchetti C , Ashing K , et al. Management of anxiety and depression in adult survivors of cancer: ASCO guideline update. J Clin Oncol. 2023;41(18):3426‐3453. 10.1200/JCO.23.00293 37075262

[38] Lydiatt WM . Prevention of depression with escitalopram in patients undergoing treatment for head and neck cancer: randomized, double‐blind, placebo‐controlled clinical trial. JAMA Otolaryngol Head Neck Surg. 2013;139(7):1. 10.1001/jamaoto.2013.3371 23788218

[39] Philteos J , Noel CW , Hallet J , Eskander A . Mental health considerations in patients undergoing complex head and neck reconstruction. Curr Opin Otolaryngol Head Neck Surg. 2022;30(5):380‐383. 10.1097/MOO.0000000000000827 35924661

